# Application of an Improved Multipoint Optimal Minimum Entropy Deconvolution Adjusted for Gearbox Composite Fault Diagnosis

**DOI:** 10.3390/s18092861

**Published:** 2018-08-30

**Authors:** Wenan Cai, Zhijian Wang

**Affiliations:** 1 College of Mechanical Engineering, Taiyuan University of Technology, Taiyuan 030024, China; caiwenan0008@link.tyut.edu.cn; 2 College of Mechanical Engineering, The North University of China, Taiyuan 030051, China

**Keywords:** ensemble local mean decomposition, combining product function, multipoint optimal minimum entropy deconvolution adjusted, joint fault feature

## Abstract

The fault feature extraction of gearbox is difficult to achieve under complex working conditions, and this paper presents a hybrid fault diagnosis method for gearbox based on the combining product function (CPF) and multipoint optimal minimum entropy deconvolution adjusted (MOMEDA) methods. First, ensemble local mean decomposition (ELMD) is utilized to reduce the noise in original signal, and get a series of product functions (PFs), through the correlation coefficient method to remove false components and residual components. Then, multi-point kurtosis of the definition is achieved by calculating the multi-point kurtosis spectrum of each layer PF, and the fault feature period is extracted and the PFs without periodic impact are removed. After that, in order to maintain the integrity of the original signal, the PFs with the same period are recombined by the combined product function method. Finally, the different cycle interval is configured, reduce the noise through MOMEDA on the combined signal, to further extract the fault feature. The method is applied to the feature extraction of gear box composite fault to verify the feasibility of this method.

## 1. Introduction

The gearbox is the most important power transmission component in mechanical equipment, and its health status directly affects whether mechanical equipment can work properly. Mechanical equipment in complex conditions may undergo two or more interrelated and mutual coupling failures at the same time. Their position and the degree of damage are generally different, coupled with background noise interference, which causes difficulties in complex fault feature extraction. When the failures occur on the inner race, outer race and rolling elements, it will produce periodic impulses during the course of use [1,2,3]. Simultaneous detection of multiple faults is still a big challenge in monitoring and diagnosis of rotating machinery conditions [4]. In recent years, empirical mode decomposition (EMD) has been widely used in the fault diagnosis of bearings and gears. Bustos applied EMD to gear fault identification in high-speed trains [5]. To alleviate mode mixing, Wu and Huang developed ensemble empirical mode decomposition (EEMD) to improve EMD [6]. The so-called modal aliasing refers to the fact that the same IMF contains a large feature time scale, or the same time scale is decomposed in different IMFs, which further leads to energy leakage. By adding noise in the original signal multiple times and calculating the average of these IMFs, the decomposition accuracy of EEMD is improved. The efficiency of EEMD is more accurate than that of EMD in mechanical fault diagnosis [7,8,9,10,11]. EEMD has an adaptive feature that can decompose complex signals into several IMFs, but noise interference inevitably leads to modal aliasing [10]. The reason is that the decomposition accuracy is greatly influenced by the applied white noise. When the selected white noise level is too large, it will lead to over-decomposition and there is still a modal aliasing phenomenon. When the selection is too small, it is not enough to change distribution of extreme points [9]. The literature [3,8,12] points out that how to adaptively select the white noise level has not yet been resolved. Wang et al. [13] optimized the white noise selection in EEMD and improved the decomposition efficiency by using the CMF method. However, through the analysis of the simulation signals and the real signals, it is still unable to avoid the phenomenon of mode mixing. In summary, EEMD has been successfully applied in the fault diagnosis, but due to improper selection of the added white noise level, there is still the phenomenon of mode mixing, resulting in energy leakage. Thus, a CMF approach is introduced so that it combines the neighboring IMFs with the same frequency band to decompose the original signal into two new combining mode function (CMFs) *Ch* and *CL* adaptively. LMD can decompose a complex signal into several PF components, each of which has a certain physical meaning [14,15]. Wang [15] used LMD and other fault diagnosis methods combined, successfully extracting the fault information, but the practice proved that when the LMD decomposition of the complex vibration signal cannot achieve satisfactory results, the main reason is that it has a modal aliasing phenomenon, the so-called modal aliasing refers to the same PF contains several time feature scales, or the same time scale is broken down in different PF, which leads to PF lost the original meaning of the original signal, and further leads to energy leakage or misdiagnosis occurred, the cause of this phenomenon is largely influenced by background noise .Based on these analysis, the noise component with the noise component and the PF with strong correlation with the original signal (including the PF produced by the modal aliasing) in the PF component is obtained by the LMD decomposition, and the noise component can be removed by the correlation coefficient. In work [16], the results showed the superiority of the LMD method by comparing with EMD method, and the results also showed that LMD method could identify gearbox work condition accurately and effectively. Reference [17] applied the LMD method to wind turbines, and as the background noise increases, the number of decomposition layers of the LMD also increases, resulting in a decrease in the decomposition efficiency of the LMD and an increase in the amount of calculation. In order to solve the effect of noise on LMD, ELMD [18] was proposed, which adds finite amplitude white noise to the original signal, and then performs LMD decomposition on the signal with white noise added. The above process is repeated several times, each time adding different white noise to the original signal, and finally all the PFs will be decomposed. The component is averaged to obtain the final decomposition result. Since the addition of white noise cannot be completely neutralized, modal aliasing cannot be completely avoided.

McDonald [19] proposed the Multipoint Optimal Minimum Entropy Deconvolution Adjusted (MOMEDA) of the rotating machinery fault extraction method, which uses non-iterative filters to find some continuous impact signals in a strong noise environment, these targets are suited for feature extraction of vibrating sources that produce a shock pulse per revolution of a rotating machine, however, when the multi-fault coexistence or in the strong background noise, due to the fault cycle more than one or the impact of background noise caused by this method is difficult to accurately extract the fault cycle components, for this need to pre-process the vibration signal, ELMD can adapt to the original signal noise reduction, although ELMD has a modal aliasing phenomenon, each layer and the original signal correlation PF has inherent time scale, the introduction of multi-point kurtosis definition, by calculating the PF multi-point kurtosis can get the impact cycle. Then different CPF can be combined with the same cycle of PF, which not only improves the energy of each fault feature but also maintains the integrity of the original signal, and can break down the different fault features in different PFs. Finally, enter the appropriate cycle interval, through MOMEDA we extract each CPF fault characteristics.

This paper explores a method of extracting fault features of gearboxes based on CPF-MOMEDA, through the double analysis of the simulation signal and the measured signal, and it identifies its fault characteristics accurately, which provides a new idea for extracting mechanical fault features. This paper is divided as follows: Section 2 introduces the basic theory of the LMD method and MOMEDA method. Section 3 proposes multi-fault feature recognition method for combining the product function (CPF)-MOMEDA. Section 4 describes the experiments and proves the validity of the above correction methods. Then, the result discussion and concluding remarks are given in Section 5.

## 2. Basic Theory

### 2.1. LMD Method

The role of LMD is to decompose a more complex signal into multiple components called physical functions (PFs). The PF component can be obtained by multiplying the envelope signal by a pure FM signal. For a certain signal *x*(*t*), the decomposition process is as follows [10]:

(1) According to all the local extreme points of the signal *x*(*t*), calculate the average value $m_{11}(k)$ and envelope estimate $a_{11}(k)$ of two adjacent extreme points.
(2) Connect all the points adjacent to the mean values with polylines, and then smooth the line by the sliding average method to obtain the local mean function $m_{11}(t)$. The envelope estimates are processed in the same way as the averages to obtain the envelope function $a_{11}(t)$.
(3) The local mean function *m*_11_(*t*) is separated in the original signal *x*(*t*) to obtain $h_{11}(t)$ and demodulated:
  (1) $$h_{11\;}(t)=x(t)-m_{11}(t),s_{11}(t)=h_{11}(t)/a_{11}(t)$$
(4) The above process is iterated to obtain an envelope estimation function $a_{12}(t)$. If $a_{12}(t)$ ≠ 1, then $s_{11}(t)$ is not a pure frequency modulated signal. The iterative process continues until $a_{1n}(t)$ = 1. The iteration steps are as follows:
  (2) $$\left\{ \begin{array}{l} h_{11\;}(t)=x(t)-m_{11}(t)\\ h_{12}(t)=s_{11}(t)-m_{12}(t)\\ \;\cdot \\ \;\cdot \\ \;\cdot \\ h_{1n}(t)=s_{1(n-i)}(t)-m_{1n}(t) \end{array}\right.$$
  where:
  (3) $$\left\{ \begin{array}{l} s_{11\;}(t)=h_{11}(t)/a_{11}(t)\\ s_{12}(t)=h_{12}(t)/a_{12}(t)\\ \;\vdots \\ s_{1n}(t)=h_{1n}(t)/a_{1n}(t) \end{array}\right.$$

In fact, when the decomposition effect is unchanged, the iteration termination condition is changed to reduce the number of iterations and the running time:

(4) $$a_{1n\;}(t)\approx 1$$

(5) Calculate the envelope signal (instantaneous amplitude function):
  (5) $$a_{1}(t)=a_{11\;}(t)a_{12}(t)\cdot \cdot \cdot a_{1n}(t)$$
(6) Determine the first PF component that is decomposed by the original signal:
  (6) $${\mathrm{PF}\;}_{1}(t)=a_{1}(t)s_{1n}(t)$$

According to the PF, the highest frequency component of the original signal can be obtained. At the same time, it is a single component AM-FM signal, $a_{1}(t)$ is its instantaneous amplitude, and the instantaneous frequency $f_{1}(t)$ is obtained through $s_{1n}(t)$:

(7) $$f_{1}(t)=\frac{1}{2\pi \;}\frac{d\left[\arccos\left(s_{1n}(t)\right)\right]}{dt}$$

(7) A new signal *u*_1_(*t*) can be obtained by removing *PF*_1_(*t*) from the original signal. The signal is used as the original signal of the above process for iterative process until the obtained signal becomes a monotonic function:
  (8) $$\left\{ \begin{array}{l} u_{1}(t)=x(t)-PF_{1}(t)\\ u_{2}(t)=u_{1}(t)-PF_{2}(t)\\ \;\vdots \\ u_{k}(t)=u_{k-i\;}(t)-PF_{k}(t) \end{array}\right.$$

Finally get such a result:

(9) $$x(t)=\sum {\;}_{p=1}^{k}PF_{p}(t)+u_{k}(t)$$

The results show that the LMD decomposition will completely retain the information of the original signal.

### 2.2. MOMEDA Method

In 1984, McDonald [20] proposed a new norm for continuous impact deconvolution and proved the deconvolution problem geometrically. The D-norm deconvolution problem can solve the filter coefficients by an exact non-iterative process. In 2016, McDonald [14] proposed Multi D-Norm (MDN), in order to deconvolve a plurality of pulse sequences with a certain period in a certain period interval. It also introduces the definition and algorithm of Multipoint Optimal Minimum Entropy Deconvolution Adjusted (MOMEDA). This algorithm is an improvement of MED and MCKD, but does not require an iterative process of the filter. The specific calculation process is as follows:

Assume that the collected vibration signal is as shown in Equation (10):

(10) y(n) =h(n)x(n)+e(n)

where *e*(*n*) is the white noise, *x*(*n*) is the impulse train, *h*(*n*) is the transfer function, and *y*(*n*) is the collected vibration signal. The essence of the MOMEDA algorithm is to find an FIR filter that resets the input signal *y*(*n*) through the output signal *x*(*n*), the definition of MDN is as follows:

(11) $$\text{MDN }(\vec{y},\vec{t})=\frac{1}{\|\vec{t}\|\;}\frac{{\vec{t}}^{T}\vec{y}}{\left\|\vec{y}\right\|}$$

(12) $$\text{max MDN }(\vec{y},\vec{t})=\underset{\vec{f}}{\max\;}\frac{{\vec{t}}^{T}\vec{y}}{\left\|\vec{y}\right\|}$$

Equation (12) is the definition of MOMEDA, which is to maximize the MDN. The expected output of impulse deconvolution is a continuous spike. The target vector $\vec{t}$ is a constant vector that is to define the location and weight of a continuous impact in a time range. For example, $\vec{t}={\left[\begin{array}{cccccccccc} 0 & \begin{array}{cccc} 0 & 0 & 0 & 1 \end{array} & \begin{array}{cccc} 0 & 0 & 0 & 0 \end{array} & 1 & 0 & 0 & \begin{array}{cc} 0 & 0 \end{array} & 1 & 0 & 0 \end{array}\right]}^{T}$. The target vector $\vec{t}$ will aim to extract three impact components in the output signal: impact position at *n* = 5, 10, 15. MDN is normalized to between 0 and 1, where a value of 1 indicates that a shock pulse has been extracted to achieve the target solution. At different sampling frequencies, it can extract different fault periods without resampling. It also has an automatic identification function, and by setting different parameters, it can identify the period of different fault characteristics at the same sampling frequency. Therefore, the target vector $\vec{t}$ can be used to determine the separation and position of the impulse signal.

Calculating the derivative of the filter coefficients ($\vec{f}=f_{1},f_{2},\ldots f_{L}$) solves the extremum of Equation (12):

(13) $$\frac{d}{d\vec{f}\;}\left(\frac{{\vec{t}}^{T}\vec{y}}{\left\|\vec{y}\right\|}\right)=\frac{d}{d\vec{f}}\frac{t_{1}y_{1}}{\left\|\vec{y}\right\|}+\frac{d}{d\vec{f}}\frac{t_{2}y_{2}}{\left\|\vec{y}\right\|}+\frac{d}{d\vec{f}}\frac{t_{3}y_{3}}{\left\|\vec{y}\right\|}+\ldots +\frac{d}{d\vec{f}}\frac{t_{N-L}y_{N-L}}{\left\|\vec{y}\right\|}$$

As we know:

(14) $$\begin{array}{l} \frac{d}{d\vec{f}\;}\frac{t_{k}y_{k}}{\left\|\vec{y}\right\|}={\left\|\vec{y}\right\|}^{-1}t_{k}{\vec{M}}_{k}-{\left\|\vec{y}\right\|}^{-3}t_{k}y_{k}X_{0}\vec{y}\\ \;{\vec{M}}_{k}=\left[\begin{array}{c} x_{k+L-1}\\ x_{k+L-2}\\ \vdots \\ x_{k} \end{array}\right] \end{array}$$

Therefore Equation (13) becomes:

(15) $$\frac{d}{d\vec{f}\;}\left(\frac{{\vec{t}}^{T}\vec{y}}{\left\|\vec{y}\right\|}\right)={\left\|\vec{y}\right\|}^{-1}\left(t_{1}{\vec{M}}_{1}+t_{2}{\vec{M}}_{2}+t_{3}{\vec{M}}_{3}+\ldots +t_{N-L}{\vec{M}}_{N-L}\right)-{\left\|\vec{y}\right\|}^{-3}{\vec{t}}^{T}\vec{y}X_{0}\vec{y}$$

Simplifying the above equation, we get:

(16) $$t_{1}{\vec{M}}_{1}+t_{2}{\vec{M}}_{2}+\ldots +t_{N-L\;}{\vec{M}}_{N-L}=X_{0}\vec{t}$$

Equation (15) is equal to 0:

(17) $${\|\vec{y}\|\;}^{-1}X_{0}\vec{t}-{\left\|\vec{y}\right\|}^{-3}{\vec{t}}^{T}\vec{y}X_{0}\vec{y}=\vec{0}$$

That is:

(18) $$\frac{{\vec{t}}^{T}\vec{y}\;}{{\left\|\vec{y}\right\|}^{2}}X_{0}\vec{y}=X_{0}\vec{t}$$

Due to $\vec{y}=X_{0}^{T}\vec{f}$ and ${\left(X_{0}X_{0}^{T}\right)}^{-1}$ we have:

(19) $$\frac{{\vec{t}}^{T}\vec{y}\;}{{\left\|\vec{y}\right\|}^{2}}\vec{f}={\left(X_{0}X_{0}^{T}\right)}^{-1}X_{0}\vec{t}$$

The multiple of the filter $\vec{f}$ is also the solution of Equation (19), so this is the root cause of highlighting multiple periodic shocks of MOMEDA. MED and MCKD require iterative filtering, but this method can avoid iterative operations completely and it does not require re-sampling like MCKD. In order to accurately extract the size of the fault period, a definition of Multipoint Kurtosis (MKurt) is introduced:

(20) $$\mathrm{MKurt}=\frac{{\left(\sum_{n=1\;}^{N-L}t_{n}^{2}\right)}^{2}}{\sum_{n=1}^{N-L}t_{n}^{8}}\frac{\sum_{n=1}^{N-L}{\left(t_{n}y_{n}\right)}^{4}}{{\left(\sum_{n=1}^{N-L}y_{n}^{2}\right)}^{2}}$$

**Figure 1:**
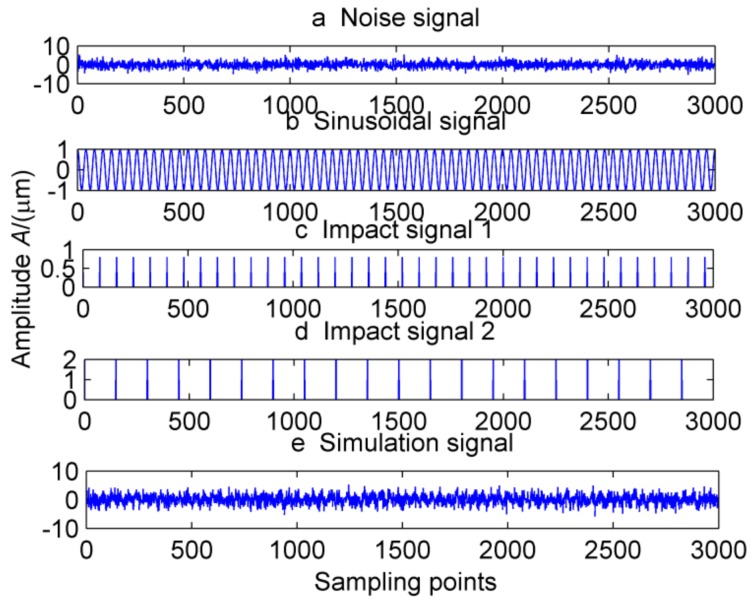
Simulation signal.

**Figure 2:**
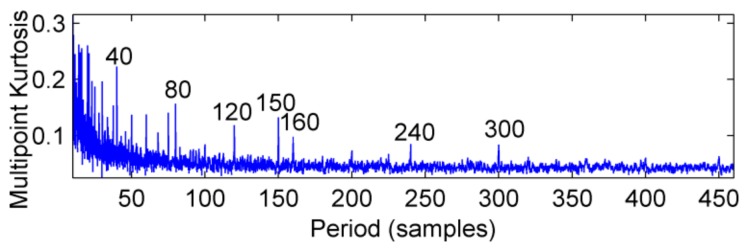
MKurt spectrum of simulation signal under low noise.

**Figure 3:**
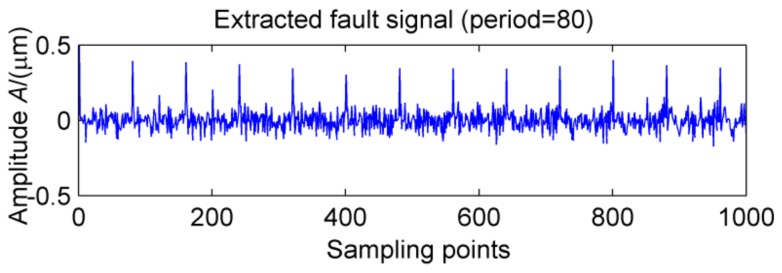
MOMEDA-filtered output impact signal at a period of T = 80.

**Figure 4:**
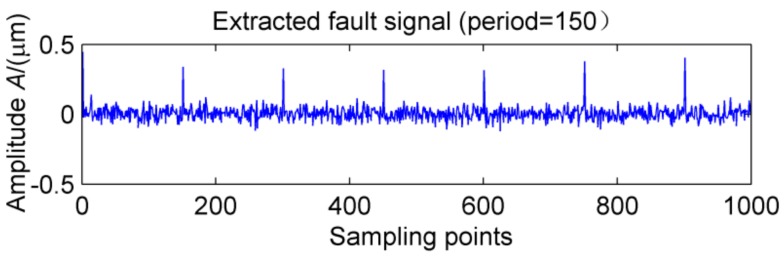
MOMEDA-filtered output impact signal at a period of T = 150.

**Figure 5:**
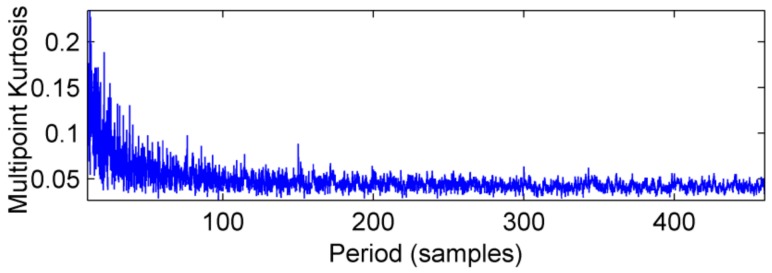
MKurt spectrum of simulation signal under strong noise.

This definition is based on kurtosis. When the target vector reaches the maximum value, a peak appears in the MKurt. The peak position is the value of the periodic pulse. When the gearbox has multiple pulses of different periods, the position where the peak occurs will increase. It should be noted that the position of the periodic impact is extended to an integer multiple or 1.5 times the period. In the entire sampling interval, except for the peak, the rest is considered as noise, so MKurt can distinguish between the fault period and the surrounding non-faulty period signal. In [14], the single fault feature of the gear is extracted. To evaluate the effectiveness of the proposed method to extract multiple faults, a typical vibration signal is simulated, which is shown in Figure 1. The simulated signal contains the noise signal (amplitude is 0.5), the sinusoidal signal, the weak impact signal 1 (amplitude is 0.8, the period is 80) and Strong impact signal 2 (amplitude is 2.0, the cycle is 150), and the above synthetic signals are shown in Figure 1e. The noise amplitude is set to less than the amplitude of the periodic impact signal, and its MKurt spectrum is illustrated in Figure 2. Obviously, the positions of the peaks are at 40, 120, 80, 160 and 240, they represent a multiple of the periodic pulse signal 1, and their peaks gradually decrease. The other impact position is at 75, 150 and 300, which represents a multiple of the period of the impact signal 2. In order to further extract the fault signal, the cycle is set in the range of [75~85] and [145~155], the step is set to 0.1, and the impact signals 1 and 2 are successfully extracted by MOMEDA, as shown in Figure 3 and Figure 4. However, this fault identification method is carried out under low noise conditions. When the noise amplitude of the above simulation signals increases (the amplitude becomes 2.0), the other signal components are unchanged, and the MKurt spectrum is shown in Figure 5. It can be seen there is no peak at periods 80 and 150, and no peak at the multiple of the period from the figure that under the influence of noise. Therefore, in a noisy environment, this method is not immune.

## 3. Multi—Fault Feature Recognition Method for CPF—MOMEDA under Strong Noise

LMD is an adaptive noise reduction method, and it can improve the noise ratio of signals, but takes the noise into account in the original signal interference. The same fault characteristics are decomposed in different PFs, resulting in energy weakened of fault characteristics, especially in the complex failure of the weak components are more likely to be submerged by noise. ELMD method is to be applied to solve modal mixing.

White noise is characterized by uniform dispersion throughout the time-frequency domain, including components of different scales. When the signal adds uniformly distributed white noise, the scale of the new signal is continuous, and the components of the original signal are projected into the associated PF by the appropriate scale reference established by the white noise. Therefore, the damaged filter characteristics of the LMD are restored by the increased white noise. A single white noise results in additional noise. However, when a large amount of white noise is added, the zero-mean characteristic of white noise can be used to offset the effect of white noise on the result.

**Figure 6:**
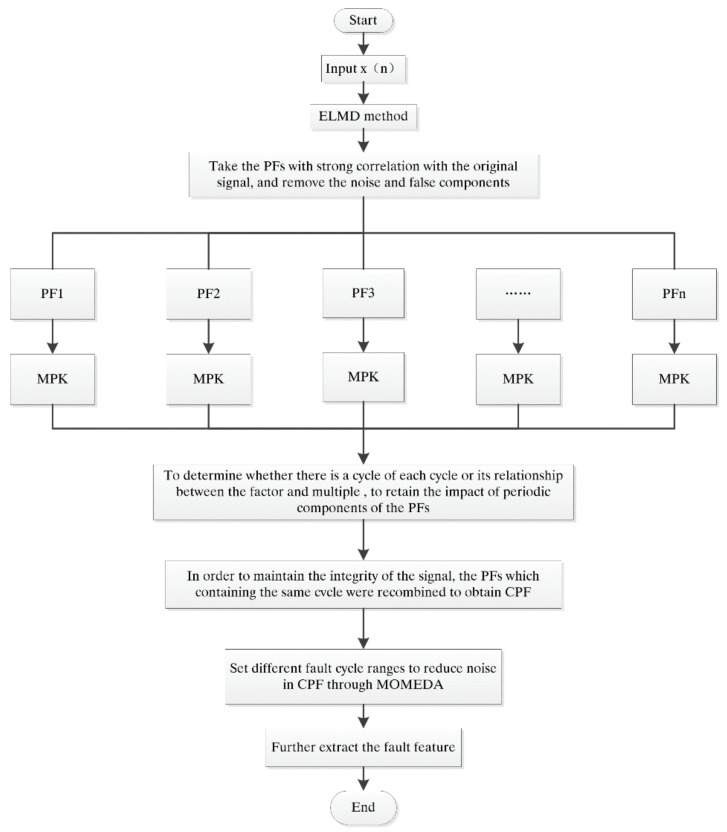
CPF-MOMEDA fault feature extraction flow chart.

To some extent, although ELMD reduces the modal aliasing phenomenon of LMD, the number of white noise added directly affects the calculation speed of LMD. In addition, ELMD still cannot attenuate the noise in the original signal completely, so modal aliasing phenomenon will inevitably continue to happen. Therefore, the energy of the same fault feature of the original signal can be enhanced by combining the product function method (CPF), and CPF is shown in Figure 6.

(1) Determine the number *M* of the overall test and the increased noise range A.
(2) Add white noise $n_{m}(t)$ with amplitude A to the original signal $x_{m}(t)$ and obtain a new signal:
  (21) $$x_{m}(t)=x(t)-n_{m}(t)\;$$
  where $n_{m}(t)$ represents the *m*-th added white noise sequence and $x_{m}(t)$ represents the noisy signal of the *m*-th trial.
(3) The additional noise signal $x_{m}(t)$ is decomposed using LMD.
(4) Repeat steps 2~3 m times, get M group PFS.
(5) Adopt the corresponding PF collection method:
  (22) $$PF_{i}(t)=\frac{1}{M}\sum_{m=1\;}^{M}pf_{im}(t)$$
  where $PF_{im}(t)$is the *i*-th PF of *m*-th trial. The result of decomposition is obtained.
(6) Get the correlation of PFs with the original signal by ELMD, preserve the strong correlation of PFs, remove the false component and noise components, and improve the signal to noise ratio of the signal.
(7) The remaining PF components are obtained for the multi-point kurtosis (MPK) spectrum, and the periodicity of the impulse signal of each layer PF is determined by the spectrum.
(8) The new CPF function is reorganized with the same period of PF, which not only enhances the energy of the same fault feature, but also decomposes the different fault features of the original signal into different PFs, this completely avoids the effect of modal aliasing:
  (23) $$CPF_{1}=PF_{1}+PF2+\ldots +PFm\;$$
  where *m* is the maximal number of PF containing the same frequency component in ELMD. CPF_2_ is obtained by combining other PF with the same periodic component:
  (24) $$CPF_{2}=PF_{m+1\;}+PF_{m+2}+\ldots +PF_{i}$$
  where *m < i ≤ n*, *n* is the maximal number of PF in ELMD, *i* is the maximum number of layers of another periodic signal. If the original signal contains three or more fault frequency components, it is possible to continue to obtain CPF_3_ or more like described above. As the ELMD adaptive signal from high to low frequency decomposition in order to get different PFs, so CPF is equivalent to an adaptive filter, it will break the original signal into high and low frequency band product function.
(9) CPF_1_, CPF_2_ contains different fault characteristics, cycle components are different, through MOMEDA extract fault characteristics. CPF-MOMEDA fault feature extraction process is shown in Figure 6.

**Figure 7:**
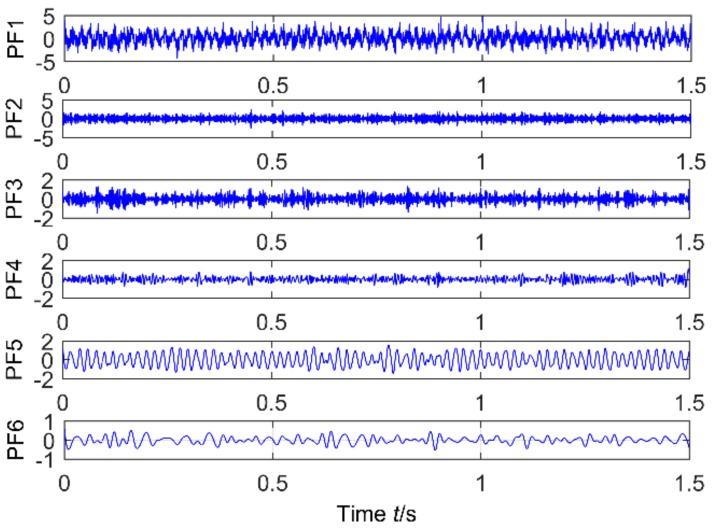
ELMD of a simulated signal in a strong noise environment.

**Figure 8:**
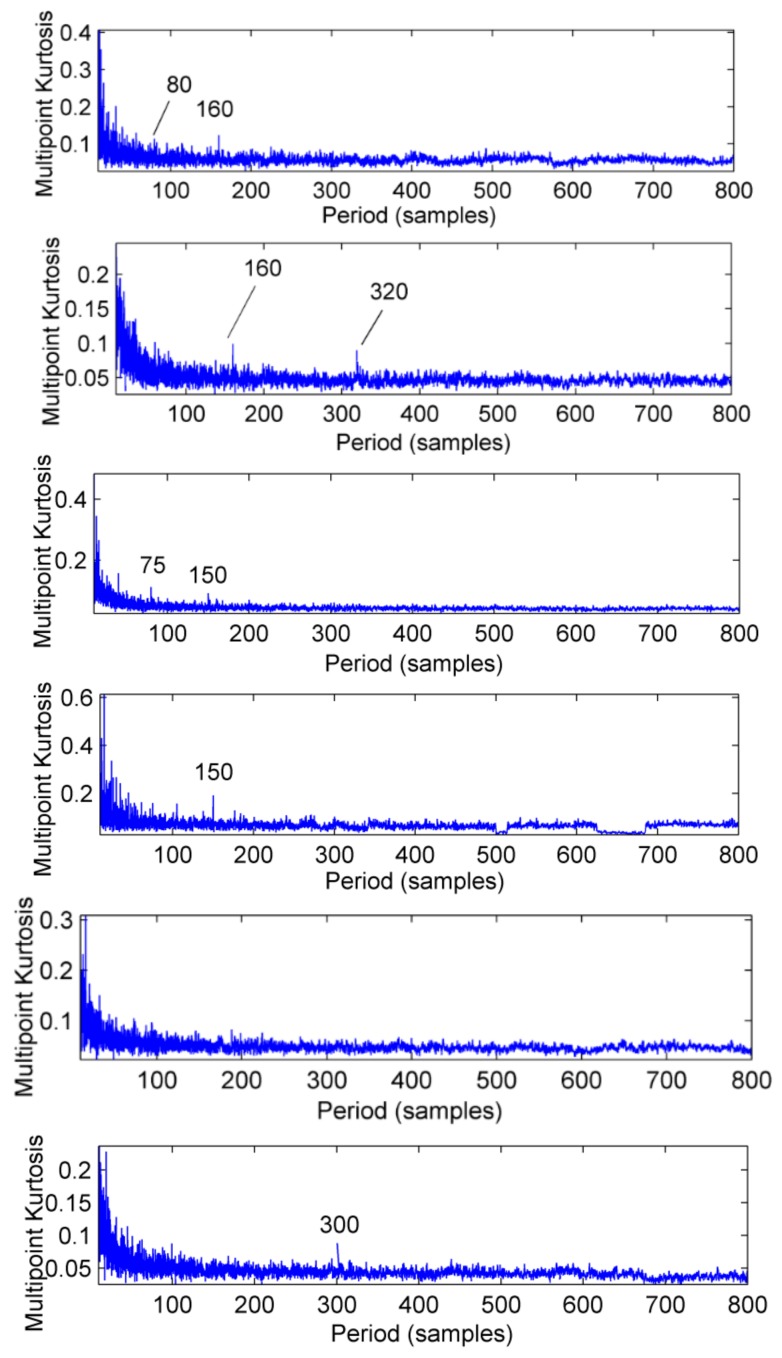
The multi-point kurtosis spectrum of the first six layers of ELMD.

**Figure 9:**
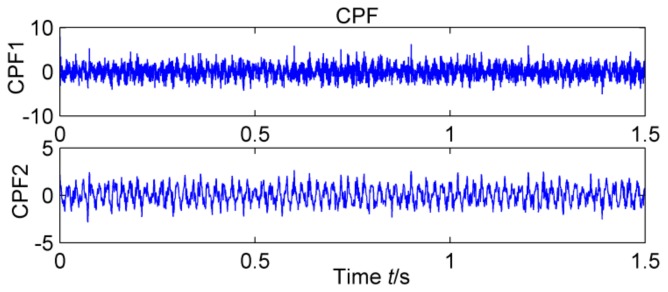
CPF_1_and CPF_2_.

**Figure 10:**
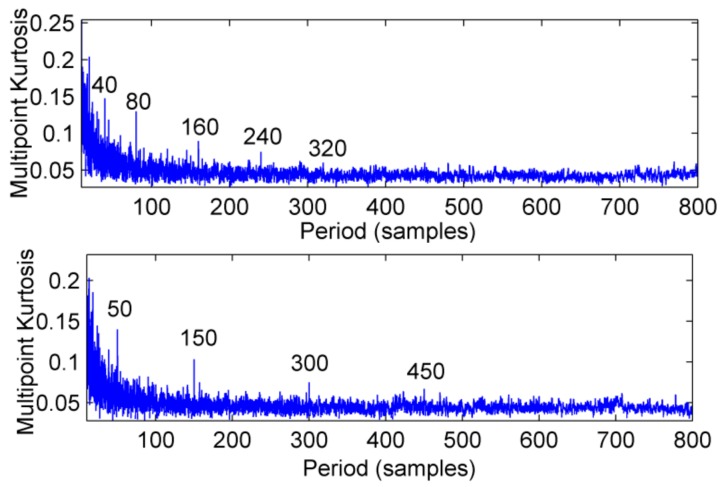
Multi-point kurtosis spectra of CPF_1_ and CPF_2_.

**Figure 11:**
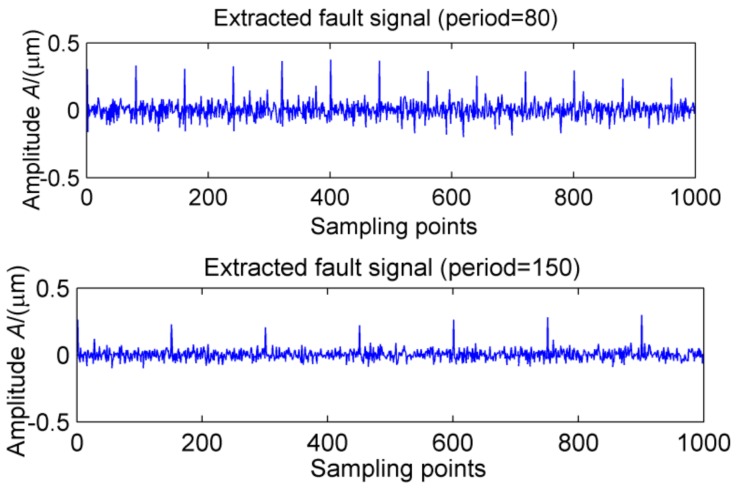
CPF + MOMEDA noise reduction signal.

The feasibility of the proposed method is verified by the simulation signal of the previous section. When the signal-to-noise ratio of the original signal is low, the composite signal is decomposed by ELMD, the first six layers PF with the strongest correlation with the original signal are shown in Figure 7, get the six-layer PF seeking more kurtosis spectrum respectively, as shown in Figure 8, the first two peaks corresponding to the cycle were 80,160,320, which is the weak impact signal 1 cycle multiple relationship; which the corresponding three-cycle components of PF_3_, PF_4_, PF_6_ were 75,150,300, which is the impact of the signal 2 factor and multiples, but in the fifth layer no peak appears, so it is further classified as a noise component. The modal aliasing phenomenon of ELMD under strong background noise is further determined by simulation signal analysis. Two impact signals are distributed in five layers of PF, and each layer peaks are relatively weak, when the noise is further enhanced, the impact signal is most likely to be submerged by noise. According to the method mentioned in this paper, which recombinant the same cycle of PF_1_ and PF_2_ into CPF_1_, recombinant the same cycle of PF_3_ PF_4_ and PF_5_ into CPF_2_. The results are shown in Figure 9. Its multi-point kurtosis results are shown in Figure 10, respectively, and obviously each layer of the impact of the cycle is very prominent, the peaks corresponding to the peaks in CPF_1_ and CPF_2_ are the multiples of the impact signals 1 and 2, respectively. The periodicity of the two impulsive pulses is determined to be 80 and 150 by multipoint kurtosis, in order to further extract the impact of the original signal components, use MOMEDA method to reduce the noise of CPF_1_ and CPF_2_ for extract the impact signal, the period interval is adjusted in the range of [75~85] and [145~155], the step length is 0.1. The results are shown in Figure 11. Apparently the impact composition is also successfully extracted, and the results are similar to those in Figure 3 and Figure 4 under low noise conditions. This further demonstrates that CPF-MOMEDA can extract complex fault shocks in strong noisy environments.

**Figure 12:**
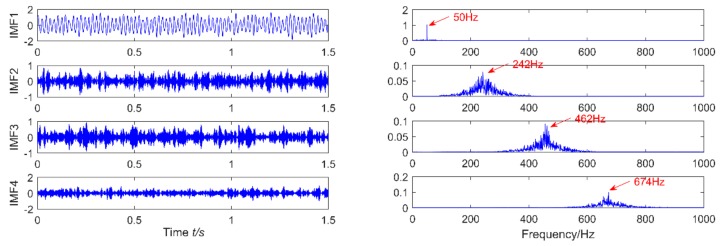
The simulation signal is decomposed using VMD results.

**Figure 13:**
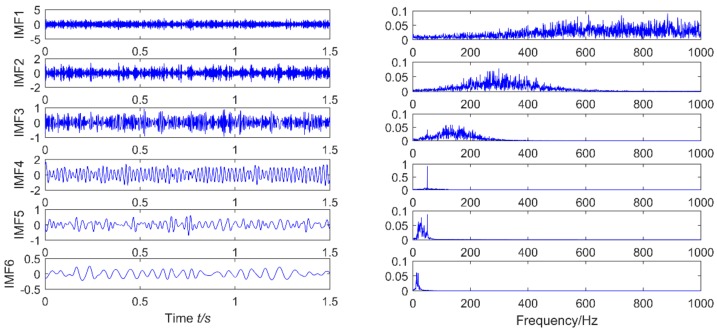
The simulation signal is decomposed using EEMD results.

In order to further compare the decomposition effects of VMD with EEMD on the impact signal, VMD can divide the original signal into several intrinsic modal functions in order from low frequency to high frequency. Each function contains a specific center frequency, but the decomposition result is affected by the two parameters which are the decomposition layer and the penalty factor. The penalty factor selected in the paper is 2000, and the number of decomposition layers is 4. The result is shown in Figure 12. It is obvious that each layer has a center frequency, but except for the first layer 50 Hz. In addition to simulating the sinusoidal component of the signal, the rest are noise components. Therefore, the VMD decomposition effect is not enough. When the simulated signal is decomposed by EEMD, the white noise amplitude is 0.2 and the integration time are 100. The decomposition result is shown in Figure 13. 

The first six layers of IMFs with the strongest correlation with the original signal are obtained. First three layers are the high-noise component. Only the fourth and fifth layers have a sinusoidal component of 50 Hz, and modal aliasing occurs. It is not immune to the EEMD of the shock signal decomposition. In addition, EEMD is a parametric decomposition method, so the decomposition error is Larger. If EEMD and VMD are selected as pre-processing methods, it is easy to cause misdiagnosis and miss diagnosis. Therefore, the article uses ELMD as a pre-processing method.

## 4. Multi-Fault Feature Recognition Experiment of Gearbox under Strong Noise Condition

**Figure 14:**
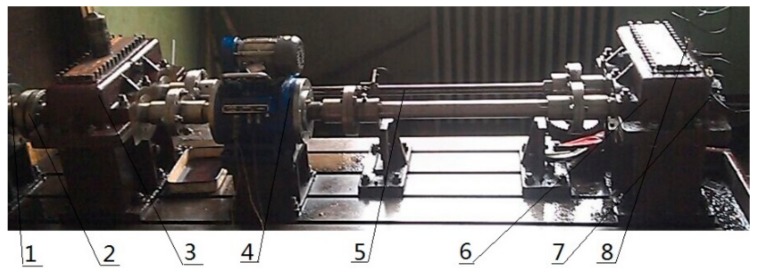
Rig for gear transmission testing. 1—Speed motor, 2—Coupling, 3—Accompanied gear box, 4—Speed reversing instrument, 5—Torsion bar, 6—Test gear box, 7—Three-way acceleration sensor 1 #, 8—Three way acceleration sensor.

**Figure 15:**
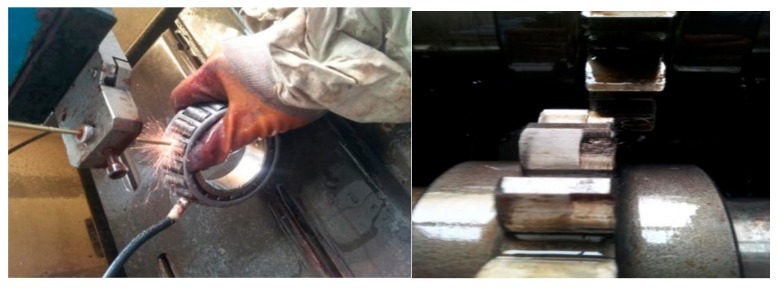
Bear and gear fault diagram.

The experimental device selected in this paper is a closed power flow experimental platform, and it is shown in Figure 14 and the loading device of the experimental platform is realized by torsion bar internal force. The experimental device mainly includes a speed display, three-way acceleration sensor, test gear (18 teeth), test bearings, motors, shafts and so on. The power of the motor is 30 kW, the constant power conversion frequency is 50–100 Hz, and the speed of the gear shaft is regulated by electromagnetic speed regulating asynchronous motor, and the range of adjustment is 120 r/min~1300 r/min. the fault types include gear peeling and bearing outer ring defects, respectively. the gear ratio of the test gear is 1:1, taking the half teeth meshing, the speed is 1200 r/min, faulty vibration signals are respectively collected for storage. The type of acceleration sensor that collects vibration signals is YD77SA, which has a sensitivity of 0.01 v/ms^2^. The z-direction data of the sensor are used for analysis. The sampling frequency is 8000 Hz, gear and bearing test torque loads are loaded to 1000 N.m, Rolling the failure frequency of 72Hz, sampling points for 2048, by simply calculating the rotation period of the shaft is 400, bearing ball failure cycle 111.1, gear meshing frequency of 360 Hz, the meshing cycle of the gear is 22.2. Wherein the gear is a peeling failure, bearing ball with EDM defects as shown in Figure 15.

**Figure 16:**
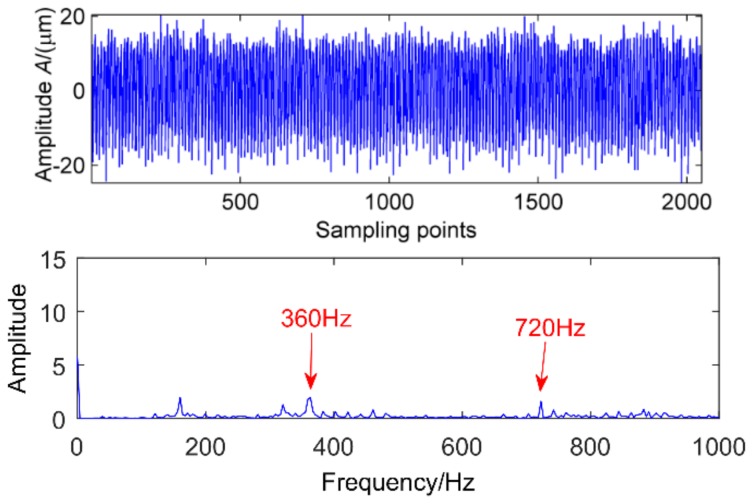
Health gear time domain waveform and fourier spectra.

**Figure 17:**
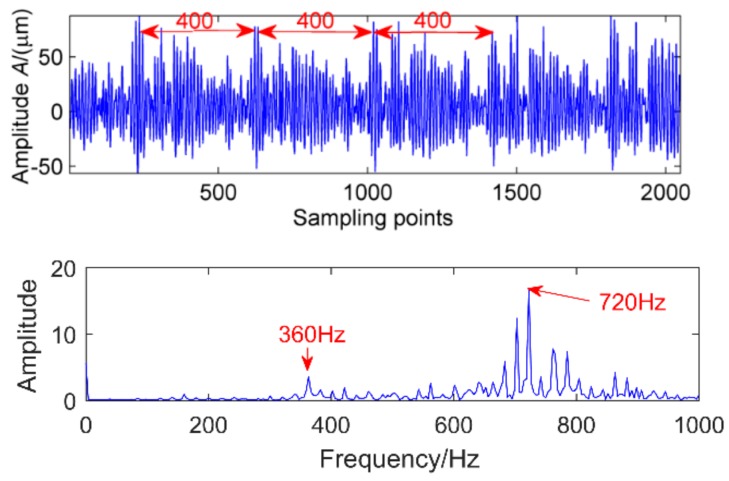
Gear pitting time domain waveform and Fourier spectra.

**Figure 18:**
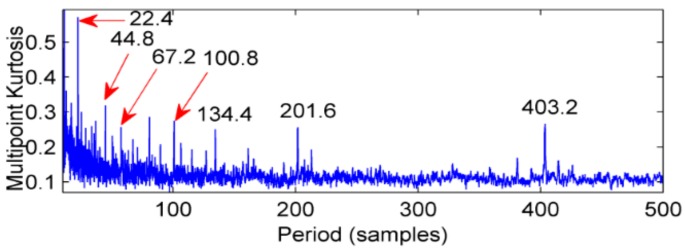
Multi-point simulation of healthy gearboxes.

**Figure 19:**
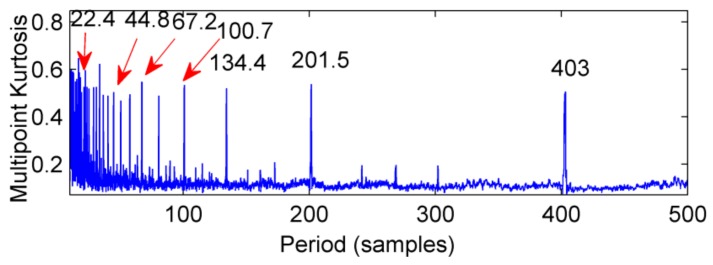
Faulty gearbox multi-point kurtosis spectrum.

The same acceleration sensor in the same direction measured gearbox health and faulty vibration signal time domain waveforms as shown in Figure 16 and Figure 17. Normal gear vibration waveform is relatively smooth, and the amplitude is small, when the gear box has failure, there will be significant shock vibration, the amplitude has increased and is basically the normal gear amplitude of about 4 times, and there are obvious periodic components appear, cycle is 400. In addition, through spectrum analysis, the amplitude of the meshing frequency of the faulty gear suddenly increases. corresponding to the rotation period of the shaft, other impact components are not definite in the time domain waveform. In order to determine the fault location, take the above two cases of multi-point kurtosis spectrum analysis respectively, the results are shown in Figure 18 and Figure 19, obviously the periods 22.4, 44.8, 67.2 and 134.4 represent the relationship between the meshing cycle of the gear and its multiple, 100.8, 201.6 and 403.2 represent the rotation period of the axis and its factors, by comparison, it can be concluded that the peak of the multi-point kurtosis of the gear is significantly stronger than that of the healthy gearbox, the peak remains at about twice. Further indicating that the gear is defective, but the bearing ball failure cycle is not extracted, indicating that the bearing failure is relatively weak, requiring further original signal noise reduction analysis.

**Figure 20:**
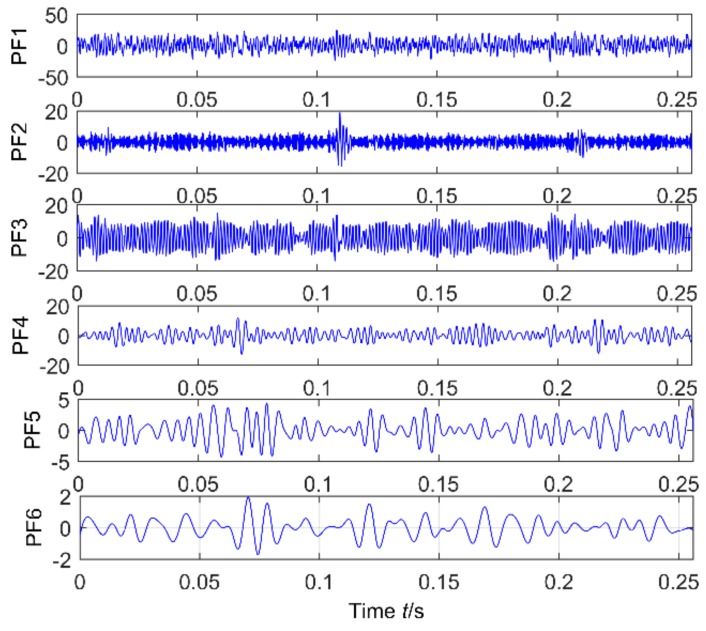
ELMD of a faulty gearbox.

**Figure 21:**
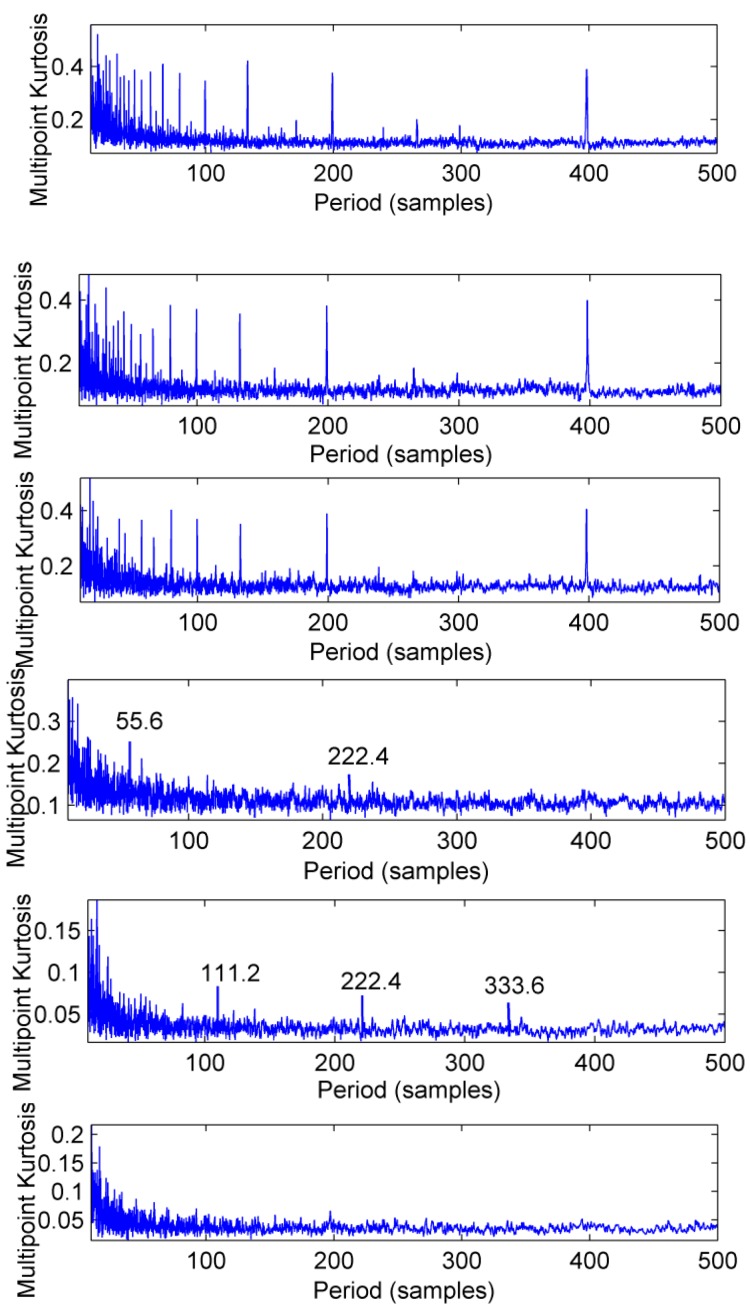
The multi—point kurtosis spectrum of the first six layers after the vibration signal ELMD is decomposed.

**Figure 22:**
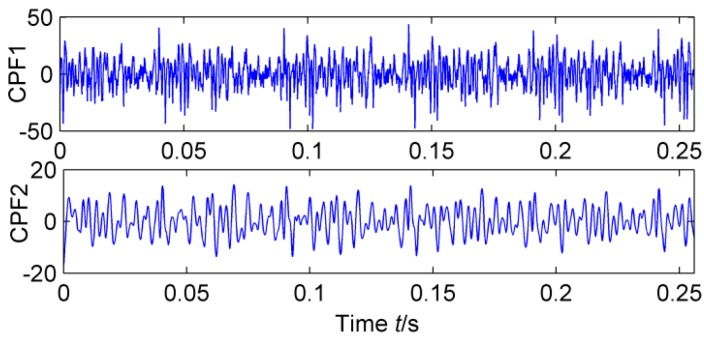
CPF of vibration signal of faulty gearbox.

**Figure 23:**
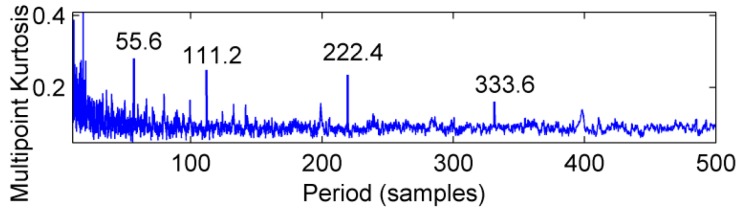
CPF_2_ multi-point kurtosis spectrum.

**Figure 24:**
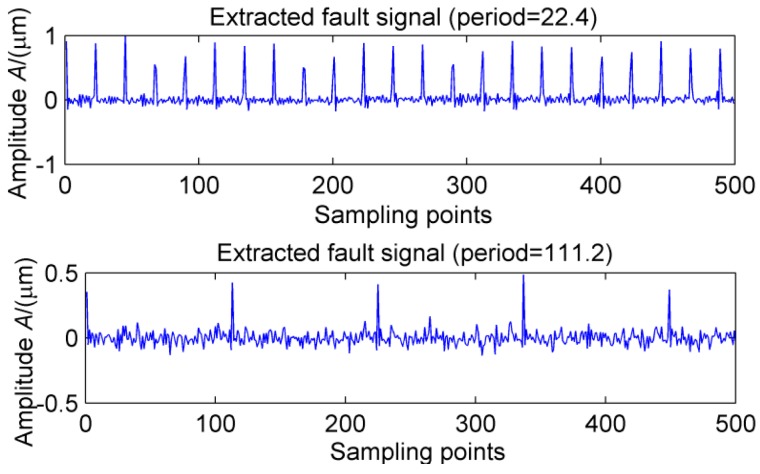
MOMEDA noise reduction for CPF_1_ and CPF_2_.

The ELMD decomposition of the vibration signal of the faulty gear box is carried out. The six-layer PFs with the strongest correlation with the original signal were obtained by the correlation coefficient method, which are shown in Figure 20. The results of multi-point kurtosis analysis of six layers of PFs are shown in Figure 21. The first three layers contain exactly the same periodic components, and they are similar to the original vibration signal, which were the rotation period of the shaft and the gear meshing cycle respectively. The first three layers contain the main signal of the main energy and fault information; PF4 and PF5 appeared to highlight another fault message, 111.2, 222.4, 333.6 represent an integer multiple of the bearing ball cycle, there is no peak in the last layer. This is a noise component and removed, in order to enhance the energy of the fault feature, CPF_1_ was obtained by combining the first three layers of PFs, the combination of PF_4_ and PF_5_ yields the CPF_2_ results, and they are as shown in Figure 22, CPF_1_ fault cycle energy is strong here do not need analysis, CPF_2_ to further solve the multi-point kurtosis, the results are shown in Figure 23, obviously the bearing fault feature is significantly enhanced. In order to further extract the impact fault characteristic signal, respectively, for CPF_1_ and CPF_2_ using MOMEDA reduce the noise, according to the bearing fault cycle and gear meshing cycle, take the cycle interval of 15–25 and 105–115, the step length is 0.1, as shown in Figure 24, extract the fault cycle component. The CPF-MOMEDA method is verified by the measured signal to have strong noise reduction performance.

## 5. Conclusions

ELMD can decompose the original signal into several product functions, but there is modal aliasing phenomenon. Therefore, the article improves the SNR by refactoring the product function. Multi-point kurtosis can determine the weak noise in the impact of the signal cycle, but in a strong background noise environment, its tracking effect is not good. MOMEDA can only extract a single fault pulse, so when there are two or more fault types, we must carry out a pre-filtering process on the original signal. The article chooses ELMD method, that can decompose different impacts of the original signal into two CPFs, which not only overcomes ELMD modal aliasing, also can improve the energy of the same shock signal. The effectiveness of CPF-MOMEDA method is proved using simulation signals and measured signals. Through this method can successfully extract complex fault characteristics, even in strong background noise, but it is also immune. In the future work, the MOMEDA method needs to be improved. How to adaptively determine the length of the filter and period is what we will study next.
